# P-1767. Understanding Antibiotic Prescribing Practices by utilizing WHO's AWaRe Criteria in a tertiary care hospital in South India: A Prospective Observational study

**DOI:** 10.1093/ofid/ofae631.1930

**Published:** 2025-01-29

**Authors:** Suresh Kumar Dorairajan, M Roshni, Suhail Hassan, Rithik Dharan, Sathya Narayanan, F Angel jenifer

**Affiliations:** Apollo hospital, Chennai, Tamil Nadu, India; Tamilnadu Dr. MGR University, Chennai, Tamil Nadu, India; Tamilnadu Dr. MGR University, Chennai, Tamil Nadu, India; The Tamil Nadu Dr. MGR Medical University, Chennai, Tamil Nadu, India; Tamilnadu Dr.MGR University, Chennai, Tamil Nadu, India; MMM Hospital, Chennai, Tamil Nadu, India

## Abstract

**Background:**

Antibiotic resistance is a significant threat globally and the inappropriate use of antibiotics leads to subsequent rise in the antimicrobial resistance causing challenge in antimicrobial stewardship programs [AMS]. Assessment of Antimicrobial use by using World health organization [WHO] categorization of Antibiotics into ACCESS, WATCH, RESERVE [AWaRe].

**Methods:**

The prospective Observational study was conducted in a tertiary care hospital in the period of one month APRIL [01.04.24 -30.04.24] using a tool containing patient’s demographic data, AWaRe classification, their indications, culture reports and prescribing practices in hospitalized patients

**Results:**

out of **1236** hospitalized patients **246** patient receive antibiotics. The results of the collected data in the following table

ANTIBIOTIC CATEGORY-

**Table:** 

ACCESS – (33.3%)		WATCH – (63.4 %)		RESERVE – (3.25%)	
DoxycyclineMetronidazole	6.9% 2.1%	Ceftriaxone Cefuroxime Piperacillin - Tazobactum Cefoperazone – Sulbactum Meropenem	28.1% 24.8% 22.4% 13.8% 7.3%	Fosfomycin Tigecycline Linezolid	0.81% 0.4% 0.4%

**Table:** 

ID REFERENCE	
Yes	26.3%
No	74.6%

**Table:** 

INDICATIONS	
Empirical	30.5%
Definitive	16.3%
Prophylaxis	48.9%
Empirical to Definitive	6

**Conclusion:**

The data indicates a predominance use of antibiotics in the "**Watch**" category, with Ceftriaxone (28.1%), Cefuroxime (24.8%), and Piperacillin-Tazobactam (22.4%) being the most commonly used antibiotics and most of the antibiotics are given Empirically (30.5%) . A quarter of antibiotics are referenced in Infectious Disease. This highlights the importance of careful antibiotic stewardship to combat antimicrobial resistance and ensure effective treatment.

**Disclosures:**

**All Authors**: No reported disclosures

